# Analysis of transcriptional responses in root tissue of bread wheat landrace (*Triticum aestivum* L.) reveals drought avoidance mechanisms under water scarcity

**DOI:** 10.1371/journal.pone.0212671

**Published:** 2019-03-06

**Authors:** Mehrdad Chaichi, Forough Sanjarian, Khadijeh Razavi, Jose L. Gonzalez-Hernandez

**Affiliations:** 1 National Institute of Genetic Engineering and Biotechnology (NIGEB), Tehran, Iran; 2 Agronomy, Horticulture and Plant Sciences Dept., South Dakota State University, Brookings, South Dakota, United States of America; Estacion Experimental del Zaidin, SPAIN

## Abstract

In this study, high-throughput sequencing (RNA-Seq) was utilized to evaluate differential expression of transcripts and their related genes involved in response to terminal drought in root tissues of bread wheat landrace (L-82) and drought-sensitive genotype (Marvdasht). Subsets of 460 differentially expressed genes (DEGs) in drought-tolerant genotype and 236 in drought-sensitive genotype were distinguished and functionally annotated with 105 gene ontology (GO) terms and 77 metabolic pathways. Transcriptome profiling of drought-resistant genotype “L-82” showed up-regulation of genes mostly involved in Oxidation-reduction process, secondary metabolite biosynthesis, abiotic stress response, transferase activity and heat shock proteins. On the other hand, down-regulated genes mostly involved in signaling, oxidation-reduction process, secondary metabolite biosynthesis, auxin-responsive protein and lipid metabolism. We hypothesized that the drought tolerance in “L-82” was a result of avoidance strategies. Up-regulation of genes related to the deeper root system and adequate hydraulic characteristics to allow water uptake under water scarcity confirms our hypothesis. The transcriptomic sequences generated in this study provide information about mechanisms of acclimation to drought in the selected bread wheat landrace, “L-82”, and will help us to unravel the mechanisms underlying the ability of crops to reproduce and keep its productivity even under drought stress.

## Introduction

Wheat (*Triticum aestivum* L.) is one of the main food crops, being consumed by humans for more than 5,000 years [1]. It provides nearly 55 percent of the carbohydrates requirements of the world population [2]. Water deficit is considered to be among the most severe environmental stresses [3] and adversely affects more than 50 percent of the wheat production area in the world [4]. Maintenance of productivity during adverse environmental conditions is one of the priority areas for plant science studies [5, 6]. Root system traits are considered to be important in maintaining plant productivity under drought stress [5, 7, 8]. Extensive and deep root systems make the plant maintain higher water potential and, therefore, have a longer period of evaporation under drought stress condition [9]. The optimal root system for drought condition is controlled by a wide range of the genes that are activated to enhance tolerance of crops to water deficiency [7, 10]. Converting this physical stress into a biochemical response will happen after the perception and recognition of the external change. Different signaling pathways are activated, each of them stimulating a set of stress-responsive genes. These signal cascades induced by a given stress finally lead to stress tolerance. On the other hand, the roots, as the first recipient of drought stress, stimulate molecular responses of the shoots through signaling cascades, allowing the plant to protect itself against water stresses [11]. It seems that plant landraces can provide genetic resources that meet forthcoming challenges for struggling with water shortage.

Landraces are defined as “a mixture of genotypes that evolved, largely by natural selection, under the environmental conditions in which they were grown” [12]. They generally have a tolerance to abiotic stresses and can produce reasonable yield under low-input farming conditions. Landraces provide a valuable gene pool for the improvement of bread wheat to be adapted to drought condition. It is imperative to study stress-responsive genes in those landraces which evolved in drought conditions [13]. Iran is considered to be a part of the center of origin [14] and genetic diversity of bread wheat (*Triticum aestivum* L.) [15]. Evaluation of these landraces will increase adaptability to drought, through the identification of genes involved in this stress [13]. Several mechanisms have been identified including drought avoidance, escape or tolerance that plants can overcome drought stress. It has been shown that plants respond to drought stress with a wide range of genes that regulate several metabolic pathways such as carbohydrate metabolism, lipid metabolism, protein folding, secondary metabolic process and Mitogen-activated protein kinase (MAPK) signaling pathway [16, 17]. A recently published review provided an overview of the candidate genes estimated that participating in drought response in plants [18]. Drought avoidance, in particular, is a strategy in which plants maintain their tissue water content relatively high, despite exposure to drought stress. In this strategy, the relatively high tissue water content is created through a deeper root system and increased hydraulic conductance, etc [19]. Despite numerous studies on drought stress in plants, regulatory networks of transcripts in response to this stress have not been completely identified, especially in the root tissue.

The availability of the next generation sequencing technologies provided high-throughput tools for studying genes expression profiles at the level of whole genome [20] that widely used for studying the plant gene response to abiotic and biotic stresses [21, 22]. A variety of studies have been performed on the transcriptional responses of wheat crop under drought stress [22–24]. Most of these studies focused on gene expression profiles of above-ground organs or well-known varieties, but not on the roots of wheat landraces. Therefore, in this research, our main objectives were to identify the genes participating in response to drought stress and investigate gene regulation networks related to drought adaptation mechanisms in the bread wheat landraces.

## Materials and methods

### Plant materials and drought treatment

Based on our previous survey of 123 Iranian bread wheat landraces, “L-82”, a spring wheat landrace was selected for this experiment due to its tolerance to drought stress. A two-stage greenhouse experimentation was conducted to evaluate these landraces for drought tolerance. The first part of the screening was executed based on the root and shoot characteristics, relative water content (RWC) and parameters derived from polyphasic chlorophyll fluorescence under drought and normal conditions. To evaluate the plant materials, a 123×2 factorial experiment in randomized complete block design with 4 replications was applied. The GGE biplot function allowed us to choose vertex genotypes close to effective traits as an indicator of water stress tolerance. Based on the results, four landraces including ‘L-118’, one from each sector, along with “Marvdasht” a well-known drought susceptible spring variety were considered for the second step of the experiment. Although the first part of the screening was done during the seedling stage of plant growth, the second stage of screening was executed at the reproductive stage. A 5 (genotype) × 2 (well-watered and drought stress) factorial experiment in randomized block design with 2 replications and five seeds per pot was used in this stage of experimentation. Similar to the first experiment, at the second stage, screening of landraces was done based on the root and shoot characteristics, RWC and chlorophyll fluorescence parameters under contrasting water regimes. Besides, seed weight, seed number and thousand kernel weight (TKW) were also applied for this stage of screening. The results of these experiments showed a great variability for all morphological and physiological traits among wheat genotypes in the evaluated germplasm. This enabled us to screen genotypes that are close to the well-known traits as an indicator of water stress tolerance. It has been shown that genotype ‘L-82’ is located close to most of the well-known traits as an indicator of water stress tolerance (i.e., Performance index for the photochemical activity, TKW, seed weight and root length). Therefore, this genotype was used for further evaluation in this experiment. “Marvdasht” (HD2172/Bloudan//Azadi), a well-known Iranian drought-susceptible variety of bread wheat [25], was used as a sensitive control in the experiment. The experiment was conducted in greenhouse conditions (16 h daylight at 25 ± 3 °C and an 8 h dark period at 17 ± 3 °C) at Hamedan, Center of Agriculture and Natural Resources Research (Iran). Each of the selected landraces was planted (five seedlings per pot) in 120-cm deep pipes with 25.4 cm diameter, filled with the same weight of field soil (33.6% sand, 34% silt and 32.4% clay). Two watering treatments were (a) control, which the soil moisture of the pots where maintained near to field capacity (FC) as calculated by Eq 1, and (b) drought stress, that the soil water content was kept at 45% FC. Each growth experiment was done with two replications. The field capacity (FC) and permanent wilting point (PWP) of the soil were measured by applying 0.3 and 15 bar pressure on the soil sample in the pressure chamber device as proposed by Abbott, 1985 [26]. The drought treatment was started by withholding water at the heading stage (Feekes’ growth stage 10.5) onwards. The soil moisture for the pots of the well-watered and drought-stressed conditions was maintained using the required amounts of water as the benefit of the handy TRIME-FM IMKO GmbH, Germany, following the manufacturer’s instructions. Pots on well-watered irrigation received water on each day, as calculated by Eq 1.

$$D_{n}=\sum_{i=1}^{m}\left[\frac{{(\theta }_{{FC}_{i}}-\;\theta_{1_{i}})\;\times \;{Bd}_{i}\;\times \;D_{i}}{{Bd}_{w}\;\times \;100}\right]$$ (1)

in which, θ_FCi_ represent the percent of moisture content at field capacity, θ_1i_ is the percent of moisture content before irrigation, Bd_i_ shows soil bulk density (g cm^−3^), D_i_ express soil depth (cm), Bd_w_ exhibit water bulk density (g cm^−3^) and D_n_ is equal to irrigation water depth (cm). Pots on drought-stressed treatment received only the corresponding fraction of the water provided to well-watered irrigation, as described above.

A total of eight samples (two genotypes × two biological replications × two irrigation regimes) were used for cDNA library construction and sequencing.

### RNA extraction, cDNA synthesis and sequencing

For RNA extraction, the root tissues of all plants in each pot were pooled and frozen immediately in liquid nitrogen and stored at -80°C. Frozen tissues were ground with a mortar and pestle and approximately 100 mg of powdered tissue was sampled. RNA was isolated using the RNA extraction kit (ZR plant RNA MiniPrep, Zymo Research, USA) according to the manufacturer’s instructions. RNA integrity and purity were assessed using a NanoDrop 2000 Spectrophotometer (Thermo Fisher Scientific, USA), an Agilent 2100 Bioanalyzer (Agilent Technologies, Santa Clara, CA, USA) and by 1% agarose gel electrophoresis. RNA samples were sent to Beijing Genomics Institute (BGI), China (https://www.bgi.com) for library construction and sequencing. Libraries were sequenced on an Illumina HiSeq 2500 generating 2x150 bp paired-end reads.

### DEGs identification and functional annotation

The sequenced reads were processed applying CLC Genomics Workbench 10.1 (CLC-BIO. Aarhus, Denmark). The raw reads were filtered by removing adapter sequences, ambiguous nucleotides and low-quality sequences. Trimming of reads were performed based on a minimum quality of Q27 and a maximum of two ambiguous nucleotides. All downstream analyses were based on trimmed data. Pearson’s correlation coefficients were applied to quantify the correlation between biological replicates.

The high-quality reads were assembled using the wheat reference genome sequences TGACv1 (Ensemble plants release 36) as a guide. The de novo assembly tool within the CLC Genomic Workbench software allowing the software to optimize the kmer size. The assembly was further refined by mapping all the reads back (Mismatch cost = 2, insertion cost = 3, deletion cost = 3, length overlap = 0.5 and similarity fraction = 0.8).

Reads from each individual sample were mapped to the transcriptome assembly using the RNA-Seq tool in CLC with the same similarity parameters used in the assembly phase detailed above. Empirical analysis of differentially expressed genes (DEGs) developed by Robinson and Smyth [27] were employed in this study to find DEGs. The test uses the raw counts and implicitly carries out normalization and transformation of these counts. This test was carried out to select differentially expressed genes by calculating the fold change of each transcript for each genotype under stress condition in comparison to their respective control. The genes having a minimum of 50 reads in at least one sample type were removed prior to differential expression analysis. The criteria to identify the putative differentially expressed transcript were false discovery rate (FDR) ≤ 0.01 and fold change (FC) ≥ 2 or ≤ -2. Statistical test was done between each genotype under water stress compared to its control.

Gene ontology enrichment was carried out using the singular enrichment analysis (SEA) function of the web-based tool AgriGO [28]. The input list consisted of different sets of DEGs, and annotation of wheat genome (Ensemble plants release 36) was used as background. Overrepresented terms in the three main categories, “Biological Process”, “Molecular Function” and “Cellular Component” were filtered using Fisher’s exact test and the Benjamini-Hochberg multiple testing corrections (Q-value < 0.05). The resulting GO terms were plotted with the Web Gene Ontology Annotation Plot (WEGO) tool [29]. Pathway enrichment analysis of DEGs was performed using the Kyoto Encyclopedia of Genes and Genomes (KEGG, http://www.genome.jp/kegg/). Heat maps were generated using the web-based tool Morpheus (https://software.broadinstitute.org/morpheus/).

### RT-qPCR validation

To validate the RNA-Seq analysis, RT-qPCR was performed on a set of eight randomly selected genes. DNase I treated RNA samples were reverse transcribed using First Strand cDNA Synthesis Kit (Thermo Scientific). Primers for RT-qPCR were designed using the Primer3Plus online software (www.bioinformatics.nl/primers3plus) and their sequences are available in S1 Table. The RT-qPCR reactions were performed with the MiniOpticon Real-Time PCR System, Bio-Rad (Hercules, CA, USA) and the SYBR Green 2x Master Mix (Ampliqon) were used to detect transcript abundance. The reaction was performed using 1.5μl of first strand cDNA, 3μl of each primer and 7.5 μl SYBR Green Master Mix in a final volume of 15 μl. Negative control was also considered for each run. Cycling programs were incubation at 95 °C for 5 min, then 40 cycles of denaturation at 92 °C for 45 s, annealing at 60 °C for 45 s and extension at 72 °C for 45 s. The specificity of all products was verified via melting curve analysis by increasing the temperature from 60 °C to 95 °C and read every 0.5 °C. Three replicates were considered for each gene. Normalization of reads was done concerning the Actin 2 as the reference gene. The relative quantitative method (2^-ΔΔCt^) was used to estimate quantitative gene expression.

## Results

### Morphological, physiological and seed yield-related traits of the evaluated genotypes

**Fig 1:**
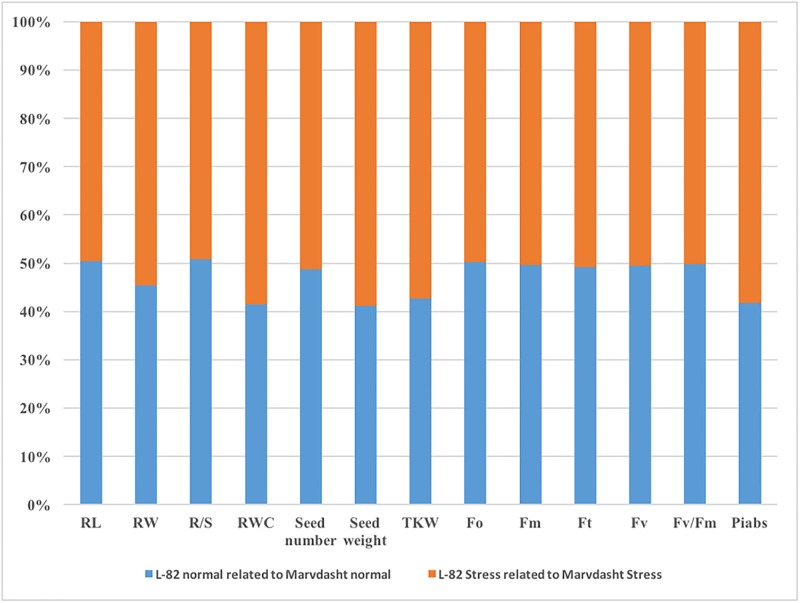
The value of each parameter in resistant landrace relative to the corresponding value of the susceptible genotype. Fo is the initial/minimal fluorescence; Fm is the maximal fluorescence value; Ft is the fluorescence intensity at time t; Fv is variable fluorescence, Fv = Fm–Fo; Fv/Fm represents the maximum quantum yield of Photosystem II (PSII); and PIabs is the performance index for the photochemical activity [30].

In this study, a landrace selected from our previous study, “L-82”, was used because of its tolerance to drought stress. The selected landrace had a deep rooting system, high root weight and high root/shoot weight ratio related to “Marvdasht”, the drought susceptible spring wheat variety (Table 1). There was a slight decrease in leaf RWC (20%) after applying drought stress treatment, while the amount of RWC decreased by ≈ 44% under the same situation. TKW, as a promising trait for increasing grain yield in wheat, was reduced by 19% after drought treatment in “L-82”. The reduction of this trait at the same water availability was much lower (40%) in susceptible genotype. Among parameters derived from polyphasic chlorophyll fluorescence, PIabs (performance index for the photochemical activity) was a very sensitive parameter to environmental stress. It has been shown that this parameter was decreased by 11% in “L-82” after drought treatment, whilst this decline was much higher (≈ 40%) in sensitive genotypes. The value of each parameter in resistant landrace relative to the corresponding value of the susceptible genotype (which thus become equal to 100%) is plotted in Fig 1. It is clear that root weight, RWC, seed weight, TKW and PIabs have been decreased in the susceptible genotype relative to resistant landrace, under drought treatment.

**Table 1:** Mean of morphological, physiological and seed yield-related traits of the evaluated genotypes under control and stress conditions.

**Genotype**	**Condition**	**Root length (cm)**	**Root weight** **(gr)**	**Root/shoot weight ratio**	**RWC** **(%)**	**Seed number**	**Seed weight** **(gr)**	**TKW** **(gr)**
L-82	Normal	99±4*	305±46	0.936±0.222	87.81±1.14	929±119	34.36±4.36	36.99±0.45
	Stress	100.5±2.5	197±11	1.57±0.047	69.62±4.06	790±20	23.64±0.66	29.93±0.14
Marvdasht	Normal	52.5±7.5	91.5±0.77	0.464±0.0013	89.2±3.47	759±153	28.55±5.14	37.78±0.85
	Stress	54±9	49±3.3	0.803±0.0737	50.15±1.87	612±98	13.77±1.46	22.70±1.25
**Genotype**	**Condition**	**Fo** ^a^	**Fm** ^b^	**Ft** ^c^	**Fv** ^d^	**Fv/Fm** ^e^	**PIabs** ^f^	
L-82	Normal	1.0401±0.0097667	5.547±0.109	283.1283333±2.475	4.5069±0.09905	0.81249±0.0020777	2.1100168±0.28893	
	Stress	1.118017±0.44983	5.291±0.201	293.895±4.3516667	4.1806±0.135933	0.7907±0.00464	1.77301±0.04226	
Marvdasht	Normal	1.018±0.0281133	5.218±0.216	253.26833±43.515	4.1996±0.1873967	0.8049±0.0024502	2.262912±0.0822166	
	Stress	1.1027±0.042933	4.917±0.143	253.595±45.93833	3.8163±0.079633	0.7761±0.006345	1.3665943±0.1025208	

* Mean of values ± standard errors of the respective traits

^a^Fo is the initial/minimal fluorescence;

^b^Fm is the maximal fluorescence value;

^c^Ft is the fluorescence intensity at time t;

^d^Fv is variable fluorescence, Fv = Fm–Fo;

^e^Fv/Fm represents the maximum quantum yield of Photosystem II (PSII); and

^f^PIabs is the performance index for the photochemical activity [30].

### Transcriptomics profiles and analysis of DEGs

As roots are the first organs to be exposed to drought, this tissue was sampled from drought-tolerant and drought-sensitive genotypes under well-watered and drought-stressed conditions. The aim was to achieve a broad survey of genes associated with drought stress in bread wheat landraces under water scarcity. Sequencing and trimming yielded 86 and 113.14 million trimmed reads from tolerant (ED, L-82 under drought and EN, L-82 under normal conditions) libraries. Likewise, 121.83 and 106.55 million trimmed reads were generated from sensitive libraries (MD, Marvdasht under drought and MN, Marvdasht under normal conditions). The sequence reads of this study are available in the NCBI Sequence Read Archive under accession number SRP140591.

**Fig 2:**
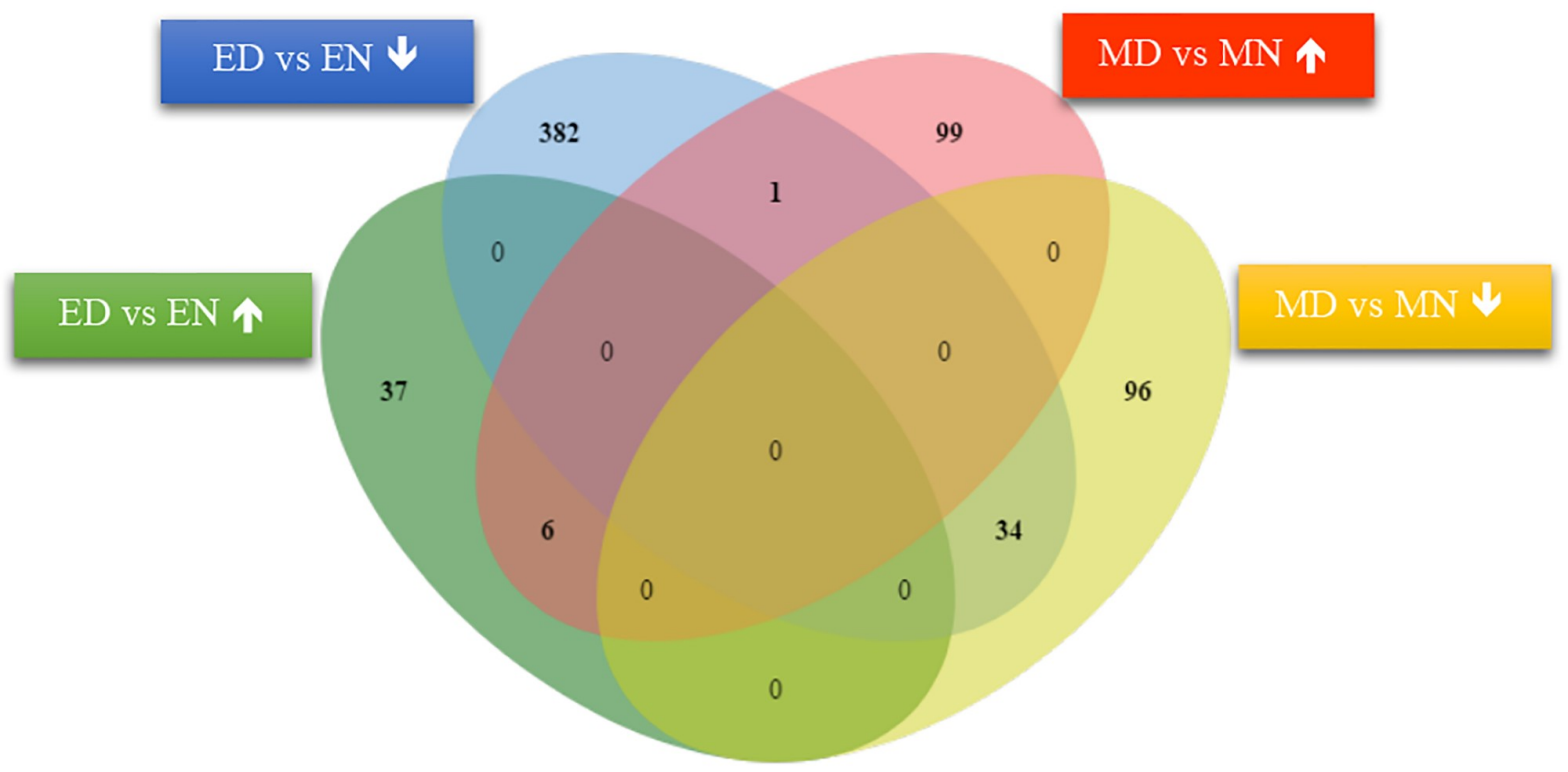
The number of DEGs (≥ 2-fold change; p ≤ 0.01) showing overlapping and specific response under various experimental conditions in drought. ED, L-82 under drought and EN, L-82 under normal conditions. MD, Marvdasht under drought and MN, Marvdasht under normal conditions. ↑ and ↓, up and down-regulated DEGs.

These reads were assembled into 118 918 transcripts representing 97 918 gene loci. Nearly, 79 percent of the reported transcripts for these libraries had a length from 500 to 1900 bp (S1 Fig). It was found that 106,681 transcripts expressed continuously in all samples. The number of expressed transcripts varied from 69.1 percent to 72.8 percent in the samples (S2 Fig). Studying of differential gene expressions was done by calculating the fold change of each transcript for each genotype under stress condition in comparison to their respective control. The criteria to identify the putative differentially expressed transcripts were false discovery rate (FDR) p-value less than 0.01 and fold change (FC) ≥ 2 or ≤ -2. In total, 3,840 transcripts representing 1,690 unique gene loci were differentially expressed under the various experimental conditions. Among these differentially expressed gene loci, 460 (43 up-regulated and 417 down-regulated) and 236 (106 up-regulated and 130 down-regulated) genes were differentially expressed in ED vs. EN and MD vs. MN, respectively. The number of DEGs showing overlapping and specific response under various experimental conditions is plotted in Fig 2. Overall, only a small number of genes were found to be common in different comparisons between genotypes.

### Functional classification of DEGs

The singular enrichment analysis (SEA), carried out with AgriGO software [28] on the 419 and 195 unique DEGs against genome reference in tolerant and susceptible genotypes, highlighted 115 and 12 GO terms, respectively (S3 and S4 Figs). GO term enrichment analysis of 37 up and 382 down-regulated unique DEGs of tolerant genotype resulted in 1 and 110 significantly enriched GO terms (S5 Fig). “Respond to stress” (GO:0006950) in the biological process (BP) category was the only GO term enriched in up-regulated DEGs in tolerant genotype. On the other hand, “secondary metabolic process” (GO:0019748), “cell volume homeostasis” (GO:0006884), “glycerol transport” (GO:0015793), “cellular water homeostasis” (GO:0009992), “polyol transport” (GO:0015791), “water transport” (GO:0006833), “organic hydroxy compound transport” (GO:0015850), “fluid transport” (GO:0042044), “water homeostasis” (GO:0030104) and “secondary metabolite biosynthetic process” (GO:0044550) in the BP category, “glycerol channel activity” (GO:0015254), “glycerol transmembrane transporter activity” (GO:0015168) and “O-methyltransferase activity” (GO:0008171) in the molecular function (MF) category and finally “intrinsic component of plasma membrane” (GO:0031226), “integral component of plasma membrane” (GO:0005887) and “mitochondrion” (GO:0005739) in the cellular components (CC) category were the most enriched GO terms among the 382 uniquely down-regulated DEGs in tolerant genotype.

GO term enrichment analysis of 99 up-regulated DEGs of susceptible genotype highlighted 13 significantly enriched GO terms but no GO term was significantly enriched among 96 down-regulated unique DEGs of susceptible genotype (S6 Fig). Calcium ion binding (GO:0005509) and hydrolase activity, acting on glycosyl bonds (GO:0016798) in the molecular function category and plasma membrane (GO:0005886) and cytoplasm(GO:0005737) in the cellular component category were some of the significantly enriched GO terms in up-regulated DEGs of susceptible genotype (S6 Fig).

**Fig 3:**
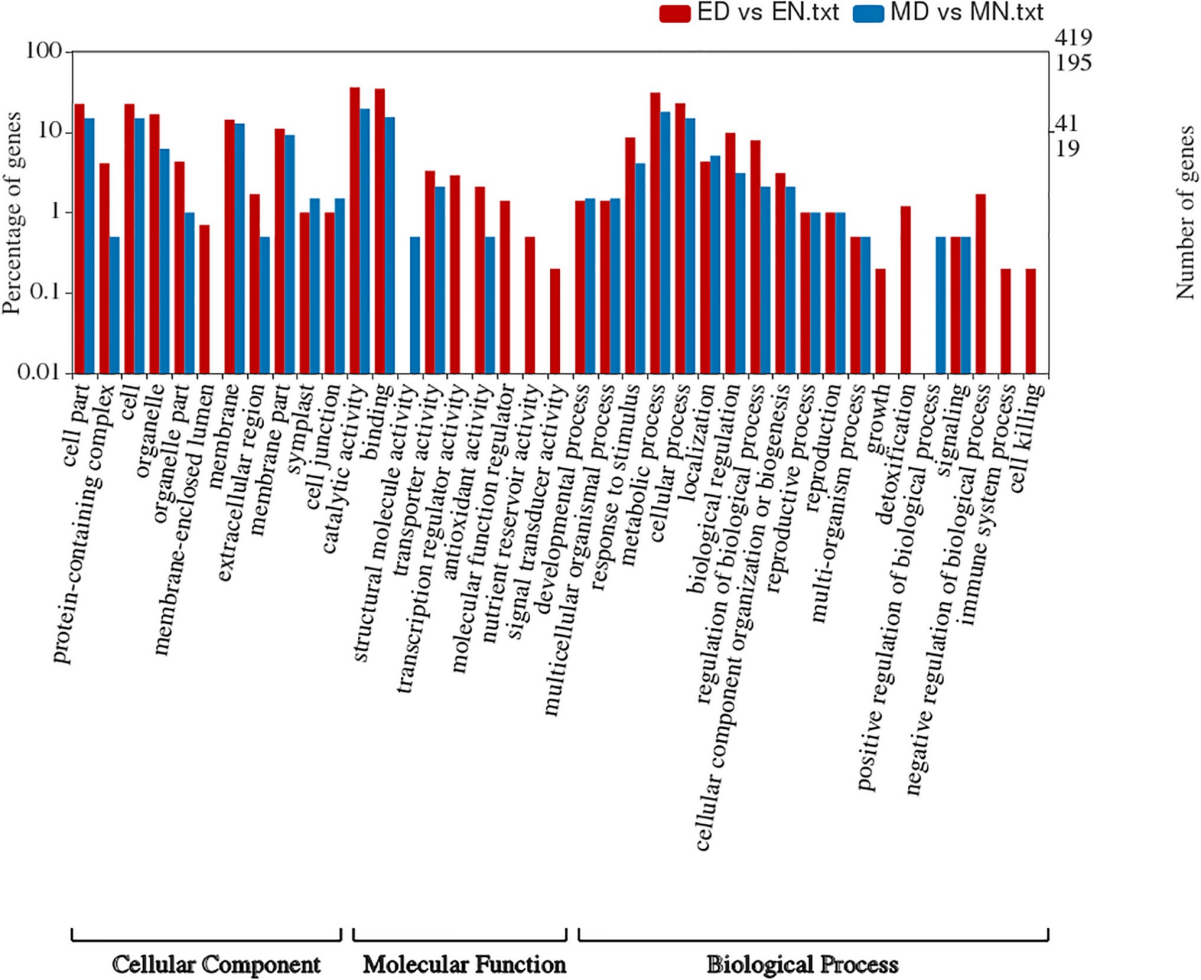
Histogram of GO terms assigned to DEGs in roots of “L-82” (n = 419) and “Marvdasht” (n = 195) in black and purple column, respectively. The DEGs are categorized into three main groups: cellular component (CC), molecular function (MF), and biological processes (BPs). ED, L-82 under drought and EN, L-82 under normal conditions. MD, Marvdasht under drought and MN, Marvdasht under normal conditions.

The entire set of DEGs was subjected to GO analysis to obtain deep functional characterization. Altogether, 419 unique DEGs (37 up and 382 down-regulated) in tolerant and 195 unique DEGs (99 up and 96 down-regulated) in susceptible genotypes were divided into 39 subcategories within three main categories including biological process (19), molecular function (9) and cellular components (11), in GO level 2. In total, 139 and 41 DEGs in the tolerant and susceptible genotypes were associated with “cellular component” terms (Fig 3). The number of DEGs associated with “molecular function” terms were 251 for tolerant and 60 for susceptible genotypes. For “biological process”, the number of associated DEGs was 182 and 51 for tolerant and susceptible genotypes (Fig 3). In both genotypes, “metabolic process”, “cellular process”, “biological regulation” and “respond to stimulus” were the most represented “biological process” subcategories. As for “molecular function”, the major subcategories were “binding” and “catalytic activity”, followed by “transporter activity” and “antioxidant activity”. “Cell” and “cell part” followed by “organelle” and “membrane” were the dominant subcategories in “cellular components” term (Fig 3). In addition, it was clear that “immune system process” (GO:0002376), “growth” (GO:0040007), “cell killing” (GO:0001906), detoxification (GO:0098754) and “negative regulation of biological process” (GO:0009892) in “biological process” term were associated with tolerant genotype DEGs (Fig 3). Besides, “transcription regulator” (GO:0030528), “molecular function regulator” (GO:0098772), “nutrient reservoir activity” (GO:0045735) and “signal transducer activity” (GO:0004871) in MF term and “membrane-enclosed lumen” (GO:0031974) in CC term were related to tolerant genotype DEGs (Fig 3).

### The most represented pathways

**Fig 4:**
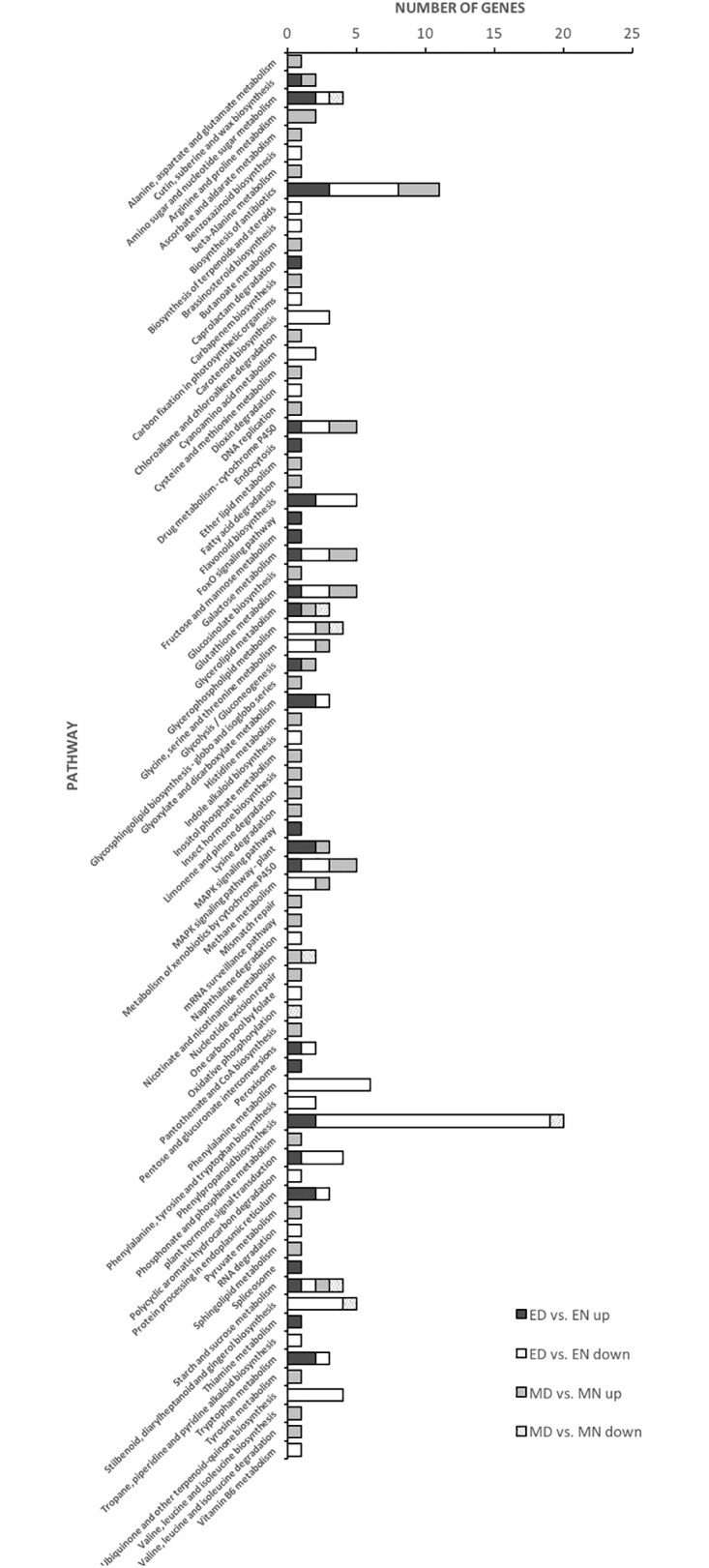
The over-represented unigenes from differential gene expression data of tolerant and sensitive libraries and their KEGG terms. ED, L-82 under drought and EN, L-82 under normal conditions. MD, Marvdasht under drought and MN, Marvdasht under normal conditions.

Having understood the functions of DEGs, we mapped all non-redundant DEGs to terms in the KEGG database and found that 47 and 46 pathways (77 KEGG pathway items) were enriched in ED vs. EN and MD vs. MN, respectively (Fig 4). These different pathways were assigned to 16 clades under four major KEGG categories, namely, “metabolism”, “genetic information processing”, “cellular processes” and “environmental information processing” (S7 Fig). “Carbohydrate metabolism”, “signal transduction” and “xenobiotics biodegradation and metabolism” were the top three up-regulated pathways represented by the uniquely expressed genes in ED vs. EN. On the other hand, the most enriched down-regulated pathways were “biosynthesis of other secondary metabolites”, “amino acid metabolism” and “xenobiotics biodegradation and metabolism” (S7 Fig).

The most enriched metabolic pathways in up-regulated DEGs of ED vs. EN were “amino sugar and nucleotide sugar metabolism”, “glyoxylate and dicarboxylate metabolism”, “MAPK signaling pathway–plant”, “phenylpropanoid biosynthesis”, “protein processing in endoplasmic reticulum” and “tryptophan metabolism”. In addition, down-regulated DEGs were enriched mostly to “phenylpropanoid biosynthesis”, “ubiquinone and other terpenoid-quinone biosynthesis”, “stilbenoid, diarylheptanoid and gingerol biosynthesis” and “phenylalanine metabolism” pathways. Additionally, most of the up-regulated DEGs of MD vs. MN were sorted to “arginine and proline metabolism, “drug metabolism-cytochrome P450”, “galactose metabolism”, “glutathione metabolism” and “metabolism of xenobiotics by cytochrome P450” pathways. On the other hand, top enriched pathways of down-regulated DEGs were “stilbenoid, diarylheptanoid and gingerol biosynthesis”, “oxidative phosphorylation” and “amino sugar and nucleotide sugar metabolism”.

All unique DEGs across different comparisons were clustered into six clades according to their expression patterns (S8 Fig). The genes in cluster I (382) down-regulated in drought-tolerant genotype while having no differential expression in control plants. On the other hand, cluster II had genes (96) that down-regulated in control plants but had no differential expression in drought-tolerant genotypes. Cluster III (35) and IV (6) comprised down and up-regulated genes with almost the same expression profile across the comparisons. Cluster V (37) exhibited DEGs which were up-regulated in drought-tolerant genotype while they had no expression in control plants. Reversely, the last cluster (VI) showed genes (99) that were up-regulated in control plants did not have not significant expression level in drought-tolerant genotype. Assessing each cluster for related GO terms showed that genes involved in “methylation”, “phenylpropanoid biosynthetic process”, “cellular detoxification” and “cinnamic acid biosynthetic process” in the biological process category and “phenylalanine ammonia-lyase activity”, “O-methyltransferase activity” and “protein dimerization activity” in the molecular function category were enriched in cluster 1 genes, which were down-regulated in drought-tolerant genotype under drought stress condition. However, genes involved in “transport”, “localization” and “response to stress” from BP category and “transporter activity” and “hydrolase activity” in MF category were of enriched GO terms in cluster 2, showing genes that down-regulated in control plants and had no DEGs in tolerant genotypes. No GO term was enriched in cluster III, IV and VI, however, “response to stimulus”, “response to stress”, “single-organism metabolic process” and “single-organism process” GO terms were enriched in cluster V, in which genes upregulated significantly in drought-tolerant genotype. The KEGG pathway analysis of each cluster was performed to assign the related biological pathways for each group of DEGs. Genes located in cluster I-VI were assigned to 31, 5, 2, 5, 16 and 37 KEGG pathways, respectively (S9 Fig).

### The most up and down-regulated genes in both genotypes

Analysis of DEGs in drought treated genotypes along with their controls will assist our understanding of the molecular events involved in drought stress response. Therefore, significantly up and down-regulated DEGs in each set of comparisons were considered in this study. Out of the 417 down-regulated genes in ED vs EN, only one gene was in common with genes up-regulated in MD vs MN; however, 34 genes were in common with genes down-regulated in MD vs MN (Fig 2). Venn diagram also showed that only 6 genes out of 43 genes were common between the up-regulated genes of ED vs EN and MD vs MN comparisons (Fig 2).

The top up-regulated DEGs in tolerant genotype under stress condition in comparison to its respective control were annotated to genes functional in secondary metabolite biosynthesis, oxidation-reduction process, heat shock proteins and chaperones, cell wall modification, transferase activity, co-factor and vitamin metabolism, DNA binding, abiotic stress response, calcium-binding domain and transmembrane transport signaling (Table 2). The most significantly down-regulated transcripts in tolerant genotype were annotated to Auxin-responsive proteins, Secondary Metabolite Biosynthesis, Ion binding, DNA binding, Protein degradation and signaling (Table 3).

**Table 2:** List of specific genes which are significantly up-regulated in ED vs. EN.

Gene ID	Fold Change	Annotation	Function
TRIAE_CS42_7AL_TGACv1_556333_AA1761120	6.29	Cinnamyl alcohol dehydrogenase	Secondary Metabolite Biosynthesis
TRIAE_CS42_1AS_TGACv1_020323_AA0076650	3.25	Cytochrome P450	Oxidation-reduction process
TRIAE_CS42_1BS_TGACv1_049748_AA0160770	2.45	Cytochrome P450	Oxidation-reduction process
TRIAE_CS42_6BS_TGACv1_513206_AA1634480	2.31	Catalase isozyme 2	Oxidation-reduction process
TRIAE_CS42_6BS_TGACv1_513206_AA1634470	2.69	Catalase isozyme 2	Oxidation-reduction process
TRIAE_CS42_4DS_TGACv1_362595_AA1180930	2.31	Heat shock protein 70	Heat shock proteins and chaperones
TRIAE_CS42_5DL_TGACv1_433530_AA1415640	3.45	UDP-glucose 4-epimerase	Cell wall modification
TRIAE_CS42_5BL_TGACv1_404728_AA1309330	3.16	Putrescine hydroxycinnamoyl transferase-like	Transferase activity
TRIAE_CS42_7DL_TGACv1_605357_AA2006220	12.15	Dolichyl-diphosphooligosaccharide—glycosyltransferase 48 kDa subunit	Transferase activity
TRIAE_CS42_3AS_TGACv1_210771_AA0678600	3.12	Fatty acyl-CoA reductase	Lipid biosynthesis
TRIAE_CS42_4DS_TGACv1_361374_AA1166440	2.78	Phosphomethylpyrimidine chloroplastic	Co-factor and vitamin metabolism
TRIAE_CS42_4AS_TGACv1_306585_AA1010680	2.91	B3 DNA binding domain	DNA binding
TRIAE_CS42_5BS_TGACv1_423362_AA1375120	2.43	Osmotin/thaumatin-like protein	Abiotic stress response
TRIAE_CS42_4AL_TGACv1_289983_AA0979890	2.13	Osmotin/thaumatin-like protein	Abiotic stress response
TRIAE_CS42_7BS_TGACv1_593723_AA1954050	3.03	Caleosin-related	Calcium-binding domain
TRIAE_CS42_6BS_TGACv1_513535_AA1644380	2.94	Bidirectional sugar transporter SWEET14-like	Probably SWEET sugar transporter
TRIAE_CS42_6AL_TGACv1_471216_AA1504800	2.14	Mechanosensitive ion channel	Transmembrane transport signaling
TRIAE_CS42_2DL_TGACv1_161880_AA0560970	5.24	Uncharacterized protein	Uncharacterized function
TRIAE_CS42_3AL_TGACv1_193635_AA0615920	4.08	Uncharacterized protein	Uncharacterized function
TRIAE_CS42_3DL_TGACv1_250209_AA0864280	2.75	Chitinase	Cell wall macromolecule catabolic process

**Table 3:** List of specific genes which are significantly down-regulated in ED vs. EN.

Gene ID	Fold Change	Annotation	Function
TRIAE_CS42_7DL_TGACv1_603709_AA1987720	-3.97	AUX/IAA protein	Auxin-responsive protein
TRIAE_CS42_1AL_TGACv1_001992_AA0037540	-2.04	Small auxin-up RNA	Auxin-responsive protein
TRIAE_CS42_1AL_TGACv1_002495_AA0042400	-8.02	Probable methyltransferase	Methyltransferase activity
TRIAE_CS42_1AS_TGACv1_019041_AA0058780	-20.29	Histone -like	DNA binding
TRIAE_CS42_2DL_TGACv1_158781_AA0526060	-4.73	Phenylalanine ammonia-lyase	Secondary Metabolite Biosynthesis
TRIAE_CS42_2AL_TGACv1_093330_AA0277780	-4.11	Phenylalanine ammonia-lyase	Secondary Metabolite Biosynthesis
TRIAE_CS42_2BL_TGACv1_129397_AA0381780	-3.17	Glycoside hydrolase	Secondary Metabolite Biosynthesis
TRIAE_CS42_4AL_TGACv1_288958_AA0962110	-3.49	Dirigent protein	Secondary Metabolite Biosynthesis
TRIAE_CS42_6BL_TGACv1_499669_AA1588820	-4.43	Serine threonine- kinase	Protein kinase activity
TRIAE_CS42_7DL_TGACv1_602629_AA1963150	-4.36	Plant peroxidase	Oxidation-reduction process
TRIAE_CS42_5AL_TGACv1_376349_AA1235590	4.04	Carotenoid hydroxylase	Oxidation-reduction process
TRIAE_CS42_2DL_TGACv1_159781_AA0542640	-5.11	Cellulose synthase	Cellulose biosynthetic process
TRIAE_CS42_1BL_TGACv1_032207_AA0126810	-4.77	Phosphoethanolamine N-methyltransferase 3	Lipid metabolism
TRIAE_CS42_5DL_TGACv1_434151_AA1430490	-3.37	NAD-dependent epimerase/dehydratase	Amino acid metabolism
TRIAE_CS42_1DS_TGACv1_080223_AA0243550	-8.07	Cysteine protease	Protein degradation
TRIAE_CS42_2BS_TGACv1_147844_AA0488140	-4.47	Aspartic peptidase	Protein degradation
TRIAE_CS42_4DL_TGACv1_342374_AA1111780	-45.93	Serine/threonine-protein kinase	Signaling
TRIAE_CS42_1DS_TGACv1_080147_AA0241250	-4.03	E3 ubiquitin- ligase RDUF1-like	Signaling
TRIAE_CS42_5DS_TGACv1_456816_AA1478520	-4.66	Aquaporin	Transport
TRIAE_CS42_5BL_TGACv1_405562_AA1330380	-3.28	NRT1 PTR FAMILY -like	Transport
TRIAE_CS42_1BL_TGACv1_030599_AA0095340	-4.07	Protein of unknown function DUF538	Unknown function
TRIAE_CS42_2BL_TGACv1_129508_AA0386470	-4.07	WAT1-related protein	Presumably transport
TRIAE_CS42_7DL_TGACv1_603849_AA1990130	-2.82	Benzyl alcohol O-benzoyltransferase-like	Transferase activity
TRIAE_CS42_5AL_TGACv1_375844_AA1227900	-17.16	Unknown	Not specified
TRIAE_CS42_3DL_TGACv1_251387_AA0881030	-2.09	Glutaredoxin-C2	Redox activity
TRIAE_CS42_2AS_TGACv1_112139_AA0331210	-2.03	Heavy metal-associated isoprenylated plant 23-like	Metal ion binding
TRIAE_CS42_7DL_TGACv1_604406_AA1997870	-10.55	Germin	Ion binding
TRIAE_CS42_2DL_TGACv1_159589_AA0540300	-2.79	Dehydrogenase/reductase	Oxidoreductases

When sensitive genotypes under drought stress were compared to control, the top upregulated DEGs were annotated to stress-responsive genes, calcium ion binding, protein degradation and transcription (Table 4). On the other hand, down-regulated DEGs in sensitive genotype were annotated to genes functional in transcription, transport, ion binding, protein degradation, lipid metabolic process and oxidation-reduction process (Table 5).

**Table 4:** List of specific genes which are significantly up-regulated in MD vs. MN.

Gene ID	Fold Change	Annotation	Function
TRIAE_CS42_7DS_TGACv1_625862_AA2065960	5.3	MYB transcription factor	Transcription
TRIAE_CS42_4BS_TGACv1_327885_AA1077740	4.06	Phosphatidylinositol 4-phosphate 5-kinase	Protein degradation
TRIAE_CS42_2BL_TGACv1_130527_AA0412950	6.04	EF-hand domain	Calcium ion binding
TRIAE_CS42_3AL_TGACv1_195082_AA0644480	3.82	EF-hand domain	Calcium ion binding
TRIAE_CS42_1DS_TGACv1_080885_AA0255140	8.73	Glycosyltransferase	Transferase activity
TRIAE_CS42_1BS_TGACv1_049649_AA0158860	10.89	AWPM-19	Response to stress
TRIAE_CS42_2BL_TGACv1_130567_AA0413710	16.51	Late embryogenesis abundant proteins	Response to stress
TRIAE_CS42_4AS_TGACv1_308636_AA1028740	2.89	Glutathione S-transferase	Oxidoreductase activity
TRIAE_CS42_4BS_TGACv1_327885_AA1077740	4.06	Phosphatidylinositol 4-phosphate 5-kinase	Signaling
TRIAE_CS42_1AS_TGACv1_019260_AA0064010	3.35	Lipase PLAT LH2	Protein binding
TRIAE_CS42_1AL_TGACv1_000553_AA0014560	4.37	Choline-phosphate cytidylyltransferase 2	Catalytic activity
TRIAE_CS42_3B_TGACv1_221059_AA0728710	5.84	Heat shock protein 70	Heat shock proteins and chaperons
TRIAE_CS42_6DL_TGACv1_526901_AA1694680	2.37	Bidirectional sugar transporter SWEET	Transport
TRIAE_CS42_2BL_TGACv1_129668_AA0392100	2.07	Niemann-Pick C1 -like	Lipid transporter activity
TRIAE_CS42_3AS_TGACv1_210771_AA0678600	2.1	Fatty acyl-CoA reductase	Lipid biosynthesis
TRIAE_CS42_6AL_TGACv1_472258_AA1520010	2.37	Annexin	Calcium ion binding
TRIAE_CS42_2AS_TGACv1_113656_AA0359020	2.37	Unknown	Not specified
TRIAE_CS42_1BL_TGACv1_032777_AA0134000	3.55	Uncharacterised protein family, basic secretory protein	Are believed to be part of the plants defense mechanism
TRIAE_CS42_6DS_TGACv1_544329_AA1747800	16.53	Not specified	Not specified

**Table 5:** List of specific genes which are significantly down-regulated in MD vs. MN.

Gene ID	Fold Change	Annotation	Function
TRIAE_CS42_6BL_TGACv1_499354_AA1579060	-4.56	bHLH transcription factor	Transcription
TRIAE_CS42_2DL_TGACv1_158876_AA0527740	-3.38	NAC transcription factor	Transcription
TRIAE_CS42_4BS_TGACv1_328328_AA1086300	-9.15	Zinc finger	Transcription
TRIAE_CS42_2AL_TGACv1_093527_AA0281800	-6.65	NAC transcription factor	Transcription
TRIAE_CS42_2BS_TGACv1_146827_AA0473530	-6.42	Protein kinase domain	Signaling
TRIAE_CS42_1DL_TGACv1_061111_AA0185720	-7.92	Subtilisin-like protease	Protein degradation
TRIAE_CS42_2DL_TGACv1_159703_AA0541750	-5.66	Serine-type peptidase	Protein degradation
TRIAE_CS42_2DS_TGACv1_177710_AA0582890	-2.65	UDP-glycosyltransferase	Transferase activity
TRIAE_CS42_6AL_TGACv1_470863_AA1497090	-19.18	Aquaporin-like	Transport
TRIAE_CS42_7AS_TGACv1_570003_AA1828470	-15.8	Phosphate transporter	Transport
TRIAE_CS42_3B_TGACv1_221341_AA0737900	-3.7	NADP-dependent malic chloroplastic	Metabolism
TRIAE_CS42_4BL_TGACv1_320461_AA1040130	-3.21	phosphatase 2C 48	Cation binding
TRIAE_CS42_3DL_TGACv1_251103_AA0877420	-9.14	Glycerophosphodiester phosphodiesterase GDPD2	Lipid metabolic process
TRIAE_CS42_1AS_TGACv1_019480_AA0067160	-5.71	Peroxidase	Oxidation-reduction process
TRIAE_CS42_4BL_TGACv1_322934_AA1073440	-9	Bidirectional sugar transporter SWEET12-like	Transport
TRIAE_CS42_2BS_TGACv1_146508_AA0467110	-3.96	WAT1-related At5g64700-like	Presumably transport
TRIAE_CS42_2BL_TGACv1_134090_AA0443680	-11.62	Germin	Ion binding
TRIAE_CS42_U_TGACv1_641430_AA2094940	-17.46	Phosphatase phospho1	Phosphatase activity

### Distribution of DEGs across the wheat genome

**Fig 5:**
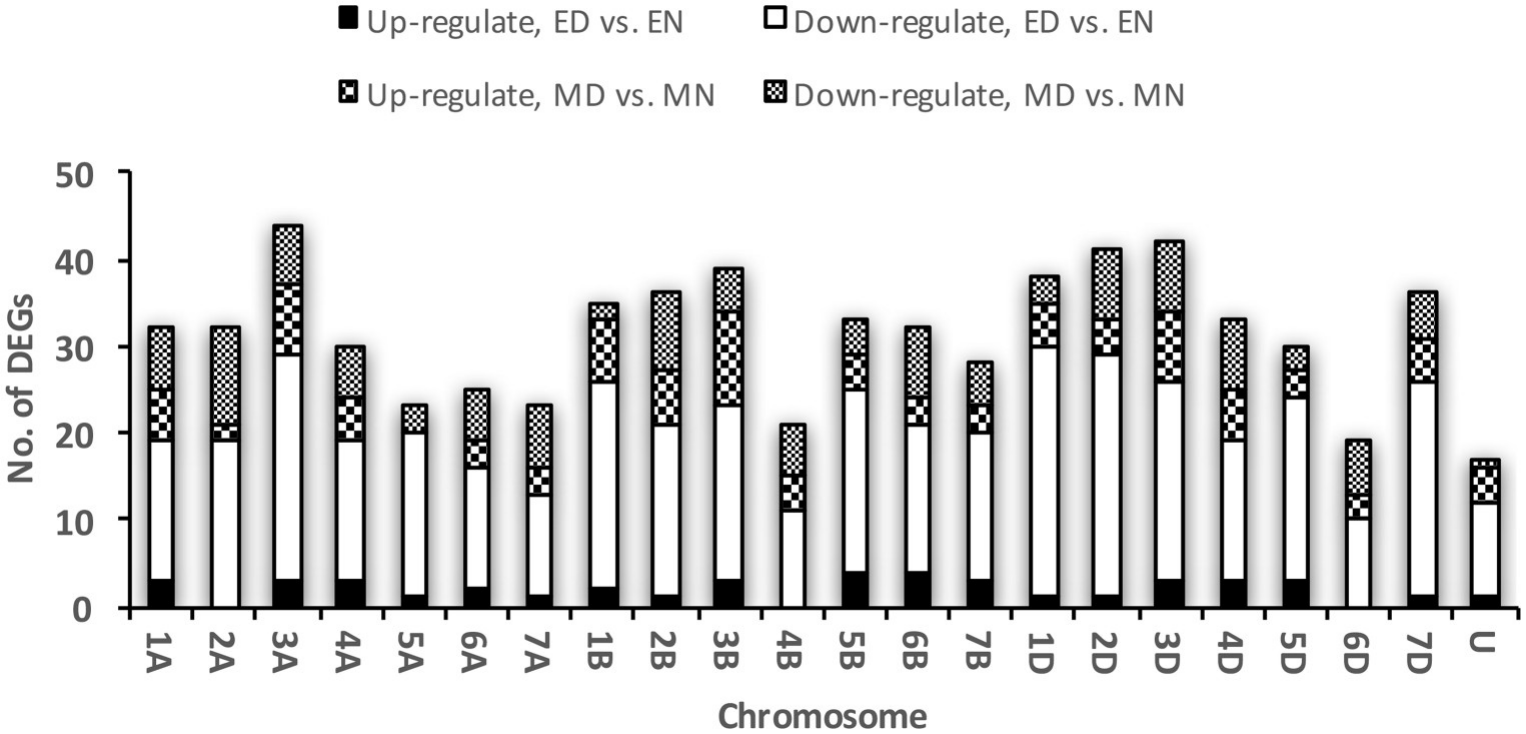
Distribution of DEGs across the wheat genome. Number of DEGs that are up or down-regulated in drought tolerance and drought sensitive genotype roots are shown in genome A, B and D. ED, L-82 under drought and EN, L-82 under normal conditions. MD, Marvdasht under drought and MN, Marvdasht under normal conditions.

The distribution of DEGs across the wheat genome showed that more up-regulated genes in drought-tolerant genotype were located in the B genome (Fig 5). However, it is obvious that all chromosomes except 2A, 4B and 6D had contributed in response to drought. In addition, most of the DEGs mapped to chromosome D were down-regulated in tolerant genotype in response to drought. On the other hand, in control plants, the most up-regulated genes were mapped to B genome while down-regulated genes in this genotype mostly occurred in A genome (Fig 5).

### RT-qPCR validation of differential gene expression

**Fig 6:**
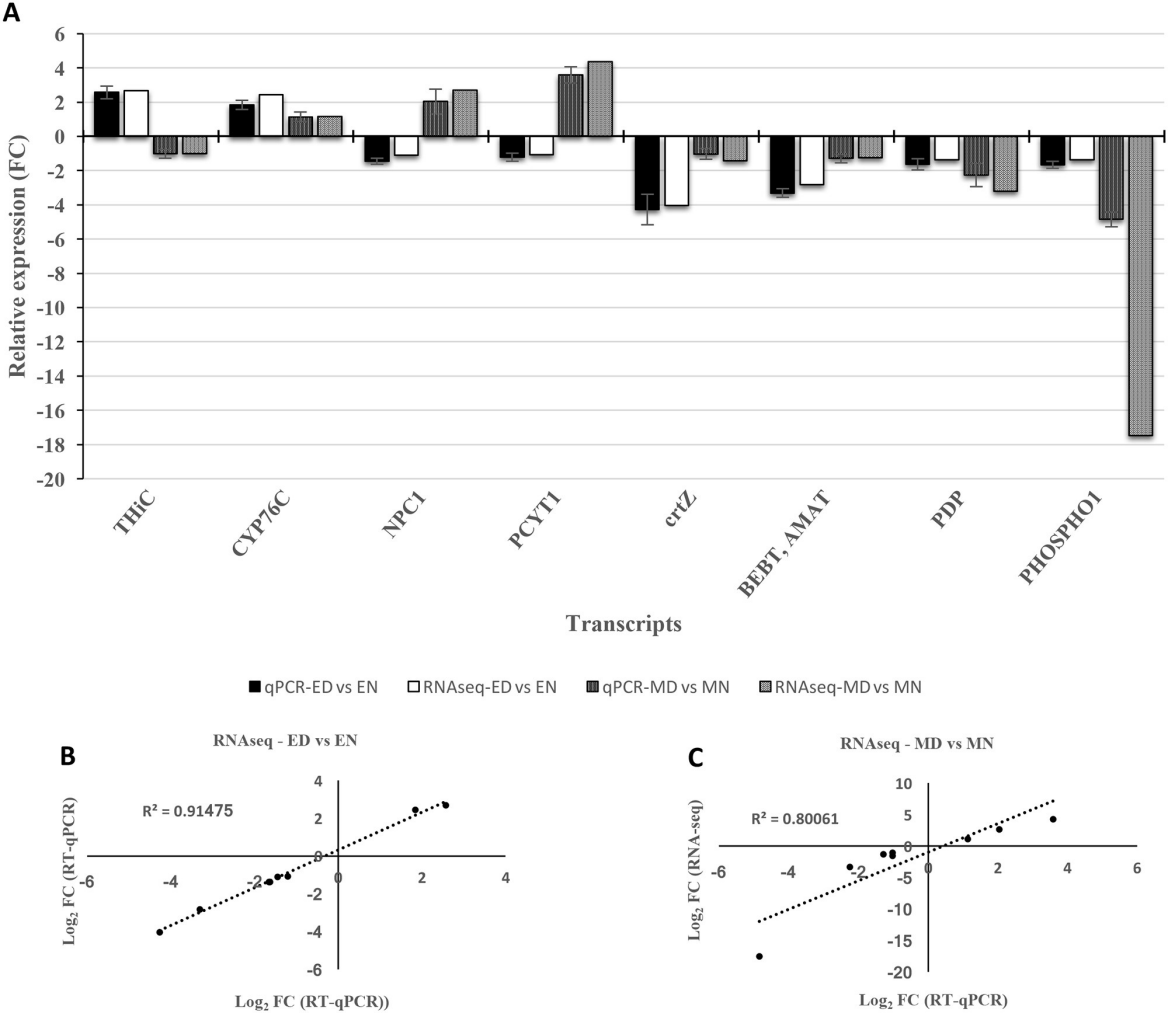
Expression patterns of 8 selected genes in “L-82” and “Marvdasht” in contrasting water regimes determined by RNA-Seq and qPCR. **RNA-Seq values represent the ratio of the expression level in drought treated genotypes to the expression level of their related controls**. For qPCR, the data are relative expression ± SD from three independent replicates (a). The qPCR primers for each contig are listed in additional file 1. Linear regression containing the RNA-Seq log_2_ value and the RT-qPCR validation date, for drought tolerance (b) and drought sensitive (b) genotypes. ED, L-82 under drought and EN, L-82 under normal conditions. MD, Marvdasht under drought and MN, Marvdasht under normal conditions.

To confirm the results of the RNA-Seq analysis, a total of eight randomly selected genes (four genes for each set of comparisons), phosphomethylpyrimidine synthase, cytochrome P450 family 76 subfamily C, Niemann-Pick C1 protein, choline-phosphate cytidylyltransferase, beta-carotene 3-hydroxylase, benzyl alcohol O-benzoyltransferase, pyruvate dehydrogenase phosphatase and phosphoethanolamine/phosphocholine phosphatase (S1 Table), were considered for RT-qPCR validation (Fig 6a). The expression level (up and down-regulated) of selected genes for qRT-PCR was the same as that in RNA-Seq except for phosphoethanolamine/phosphocholine phosphatase. However, a similar trend was apparent in the expression of this gene in both assays, but the level of expression in qRT-PCR was ≈ ¼ of that in RNA-Seq. Totally, the results illustrated that the expression data of the selected DEGs in qRT-PCR correlate highly to that in RNA-Seq analysis (Fig 6b and 6c), indicating the reliability of the transcriptomics profiling data.

## Discussion

Exposure to drought elicits a range of responses in plants that involve a large number of pathways related to different mechanisms [16, 31]. In this experiment, we evaluated differential expression of genes related to drought stress in the root tissue of wheat crop. The responses of sensitive genotype (Marvdasht) were different from tolerant landrace (L-82) in case of gene expression level and pathways involved in response to drought. However, the number of DEGs was higher in tolerant genotype but most of these genes (≈ 90%) have been down-regulated (Fig 2). On the other hand, the number of up and down-regulated genes is almost equal in sensitive genotype. Based on this finding, we hypothesize that the reaction of “L-82” happened at the very initial stages of drought stress [11]. However, the up-regulation of genes related to “secondary metabolic process” (GO: 0019748) under well-watered condition in the “L-82” affirms its intrinsic tolerance against stresses [7, 32]. The only enriched GO term in up-regulated DEGs of drought-tolerant genotype was response to stress (GO:0006950), mostly oxidative-reduction to protect the plants against oxidative stress [10, 11, 33]. The “first line of defense” against ROS and oxidative stress in plant cell is hypothesized to be antioxidant enzymes [32, 34].

### Transcriptomic profiling of tolerant landrace revealed avoidance as the dominant mechanisms in “L-82” against drought stress

Up-regulation of genes participating in phenylpropanoids (TRIAE_CS42_7AL_TGACv1_556333_AA1761120) and flavonoids (TRIAE_CS42_1AS_TGACv1_020323_AA0076650 and TRIAE_CS42_1BS_TGACv1_049748_AA0160770) pathways was observed in L-82 landrace after drought treatment. Genes coding for these secondary metabolites have the potential to protect plants against oxidative stress [34–36]. The phenylpropanoid pathway involved in the biosynthesis of different products such as lignin is critical in plant adaptation to environmental adversities [37] such as drought stress [38]. Reinforcement of cell walls for mechanical strength and for water conductance will occur with increasing of lignin content [38, 39]. On the other hand, down-regulation of lignin production as a product of phenylpropanoid compound (TRIAE_CS42_2DL_TGACv1_158781_AA0526060 and TRIAE_CS42_2AL_TGACv1_093330_AA0277780) may stimulate growing of roots [40]. It means that not only is the ability of plants to access water from deep under the soil important [19], but the roots also need to have the adequate hydraulic characteristics to allow water uptake conditions of scarcity [41]. This could be due to a differential cellular response of root tissues to conform the shape of the root as suggested by previous studies [42]. The importance of scavenging of ROS generated during drought stress has been suggested as the reason for up-regulation in flavonoids biosynthesis [43, 44] and these may play a role as antioxidants in “L-82”. Although there are contrasting reports about up or down-regulation of flavonoids in susceptible and tolerant genotypes [32, 43], it has been shown that the accumulation of these enzymes can be specific to species, genotype, stress duration and severity [32, 45]. Having considered the above results, we hypothesize that the drought tolerance in “L-82” could be due to avoidance mechanisms like optimization of water uptake [46]. Morphological assessments of this genotype under drought condition also revealed that this genotype produced strong root system under drought condition (Table 1).

However, some other aspects of drought-tolerant mechanisms like oxidative stress defense (TRIAE_CS42_6BS_TGACv1_513206_AA1634480 and TRIAE_CS42_6BS_TGACv1_513206_AA1634470) are obvious in “L-82” against drought stress [47]. Considering avoidance as the dominant mechanisms in “L-82” against drought stress, we speculate that the high number of down-regulated genes in this resistance line is related to the management of energy resources driving growth and development in response to environmental perturbations [48].

Down-regulation of genes associated with indole-3-acetic acid (IAA) (TRIAE_CS42_7DL_TGACv1_603709_AA1987720 and TRIAE_CS42_1AL_TGACv1_001992_AA0037540) was also observed under drought condition in “L-82”. It has generally been shown that auxin has a negative effect on drought adaptation in plants [19]. Down-regulation of IAA was shown to be associated with drought tolerance in plants [49]. A QTL study also showed that the up-regulation of the DEEPER ROOTING gene in rice resulted in drought avoidance and was negatively regulated by auxin [50].

Pathway analysis also showed that thiamine metabolism was up-regulated in “L-82” (TRIAE_CS42_4DS_TGACv1_361374_AA1166440) as a result of drought treatment, which was validated by RT-qPCR (THIC). Accumulation of thiamin in plants subjected to abiotic stress condition is reported by several studies [51, 52]. Interestingly, it is proved that exogenous thiamin treatment in Arabidopsis improves root growth of seedlings subjected to oxidative stress [53]. It is obvious that most of the mechanisms conferring drought resistance to “L-82” are related to root growth enhancement in which more access to water is possible through root penetration deep into the soil.

Drought stress causes endoplasmic reticulum (ER) protein folding machinery, which reaches a limit and the demand for protein folding exceeds its capacity. Increasing unfolded or miss-folded proteins in the ER triggers an unfolded protein response. This results in up-regulating the expression of genes encoding components of protein folding machinery or the ER-associated degradation system [54]. The same was detected in “L-82” (TRIAE_CS42_4DS_TGACv1_362595_AA1180930) as the expression of genes encoding heat shock protein 70 which is the most abundant chaperone protein in the ER and oligosaccharyl transferase complex was significantly up-regulated [54].

Elevated root suberin (TRIAE_CS42_3AS_TGACv1_210771_AA0678600) is also activated in both drought-treated genotypes. However, the fold change in “L-82” was almost 1.5 times more than that in sensitive genotype. It has been shown that root suberin in Arabidopsis is associated with “a root-dependent increase in time to wilting during water stress” [55]. The researchers supposed that resistance to wilting could be achieved through a reduction in water loss to the environment or via an increase in the ability to take up water from the soil. They also established that this delayed wilting was a root-dependent phenomenon [55].

Up-regulation of chitinase (TRIAE_CS42_3DL_TGACv1_250209_AA0864280) and UDP-glucose 4-epimerase (UGE) (TRIAE_CS42_5DL_TGACv1_433530_AA1415640) as two enzymes participating in “amino sugar and nucleotide sugar metabolism” pathway happened in “L-82” under drought stress. There are several observations suggesting that the UGE enzymes play a role in plant root development and/or growth [56, 57]. A possible function of UGE in plant root growth after its increased expression under drought stress is root thickness [56]. In plants, it has been observed that levels of chitinases are regulated by biotic and abiotic stress such as pathogen infection, drought, cold, salt, heavy metals and plant hormones [58, 59]. The role of chitinases has been also studied in grasses such as rye in response to cold and drought stress [60]. It has been shown that chitinases induced in tomato plants make them tolerant to drought in comparison to the susceptible genotype [61].

### B and D-genomes had a greater contribution in response to drought stress than A-genome

In our study, most of the up-regulated genes in “L-82” belong to the B genome (Fig 5). Diploid genomes A and B (what is known belong to *Triticum urartu* and *Aegilops speltoides*) formed the tetraploid wheat *Triticum turgidum* through an alloploydization event during the evolution processes. Following this step, with the contribution of the D genome (*Aegilops tauschii*), hexaploid wheat (*Triticum aestivum*) appeared [62]. However, bread wheat has a hexaploid genome but it tends towards functional diploidy [63]. Moreover, it has been shown that B-genome homoeoloci have more tendency for contribution in gene expression than the other genome homoeoloci [63]. In another study, it has been revealed that chromosomes 3B, 5B and 2B have more transcripts in both roots and leaves in response to drought stress [64]. However, most of the down-regulated genes in “L-82” belong to D genome but there are some genes that up-regulated significantly in this genome in response to drought stress, especially 3D, 4D and 5D chromosome. It have been shown that D genome of *Aegilops tauschii* has the potential of increasing drought adaptation in hexaploid wheat [65, 66]. Therefore, despite the occurrence of more down-regulated genes of this genome in root tissue of drought-treated landrace than in the other genomes, we observe that the genes that contributed in drought tolerance/avoidance belong to this genome.

In summary, this study utilized RNA-Seq data in bread wheat genotypes with contrasting drought tolerance appearance. In this study, an Iranian bread wheat landrace was used which is highly adapted to the adverse environmental condition. A review of the differential expressed genes revealed that this landrace uses the avoidance mechanism for resistance to drought. Considering this approach, the high number of down-regulated genes in resistance line probably is to manage energy resources for driving growth and development under drought stress condition. In this study, the increased gene expression in roots after drought stress was mostly associated with increasing root length in the resistant genotype, presumably part of an adaptive response maintained under intense environmental pressure. This study is a starting point to explore the network of regulatory mechanisms in Iranian wheat landraces under drought condition.

## Conclusion

The response of landraces to environmental stresses has been always an interesting topic for research given the high adaptability of these genotypes to the inappropriate environmental condition. The present study provided an opportunity to investigate the differential expression of root transcripts of bread wheat landraces under contrasting water regimes. RNA-Seq based transcriptomic analysis was applied to find out drought-responsive genes in resistant (L-82) and susceptible (Marvdasht) genotypes.

Morphological and physiological assessments point out that resistant genotype had more adaptability to drought than susceptible genotype. The first observations of transcriptome analysis showed that a large number of DEGs were down-regulated in the resistant genotype. Up-regulation of genes participating in phenylpropanoids and flavonoids pathways as well as other genes associated with oxidative stress defense was observed in resistant landrace after drought treatment. Up-regulation of chitinase, UDP-glucose 4-epimerase, root suberin, thiamine metabolism and Down-regulation of genes associated with indole-3-acetic acid in drought-treated L-82, all are related to root growth enhancement in which more access to water is possible through deep root penetration into the soil. Increasing the expression of genes associated with root growth, management of energy resources through down-regulation of a large number of DEGs, and simultaneously scavenging of ROS generated during drought stress, altogether affirmed that the drought tolerance in “L-82” could be due to avoidance mechanisms. The results of RNA-Seq analysis were confirmed by RT-qPCR assay in both genotypes under contrasting water regimes. These results indicated that this landrace could be used as a donor of appropriate genes to improve the bread wheat varieties for drought resistance.

## Supporting information

S1 Table List of primers for real-time PCR and their related sequences. (XLSX)

S1 Fig Length distribution of transcripts from wheat libraries derived from RNA-Seq data. (TIF)

S2 Fig Percentage of expressed transcripts in each sample. E and M represent “L-82” and “Marvdasht” genotype, respectively. D and N means drought stress and normal condition.
(TIF)

S3 Fig Enriched gene ontology (GO) categories in DEGs of ED vs. EN comparison (Tolerant genotype). DEGs were analyzed using AgriGo and overrepresented terms in the three main categories “Biological Process”, “Molecular Function”, and “Cellular Component” were filtered using Fisher’s exact test and the Benjamini-Hochberg multiple testing correction (Q-value < 0.05).
(TIF)

S4 Fig Enriched gene ontology (GO) categories in DEGs of MD vs. MN comparison (Sensetive genotype). DEGs were analyzed using AgriGo and overrepresented terms in the three main categories “Biological Process”, “Molecular Function”, and “Cellular Component” were filtered using Fisher’s exact test and the Benjamini-Hochberg multiple testing correction (Q-value < 0.05).
(TIF)

S5 Fig Enriched gene ontology (GO) categories in down-regulated DEGs of ED vs. EN comparison (drought tolerance genotype). DEGs were analyzed using AgriGo and overrepresented terms in the three main categories “Biological Process”, “Molecular Function”, and “Cellular Component” were filtered using Fisher’s exact test and the Benjamini-Hochberg multiple testing correction (Q-value < 0.05).
(TIF)

S6 Fig Enriched gene ontology (GO) categories in up-regulated DEGs of MD vs. MN comparison (drought sensitive genotype). DEGs were analyzed using AgriGo and overrepresented terms in the three main categories “Biological Process”, “Molecular Function”, and “Cellular Component” were filtered using Fisher’s exact test and the Benjamini-Hochberg multiple testing correction (Q-value < 0.05).
(TIF)

S7 Fig Mapping all non-redundant DEGs to terms in the KEGG database and found 47 and 46 pathways were enriched in ED vs. EN and MD vs. MN, respectively. These different pathways were assign to 16 clades under four major KEGG categories namely, “metabolism”, “genetic information processing”, “cellular processes” and “environmental information processing”.
(TIF)

S8 Fig Clustering all unique DEGs across different comparisons were categorized them into six clades according to their expression patterns. The genes in cluster I (382) down-regulated in drought tolerance genotype while had no differential expression in control plants. Cluster II had genes (96) that down-regulated in control plants but have not differential expression in drought tolerant genotypes. Cluster III (35) and IV (6) comprised down and up-regulated genes with almost the same expression profile across the comparisons. Cluster V (37) exhibited DEGs which are up-regulated in drought tolerance genotype while had no differential expression in control plants. Cluster VI shows genes (99) that are up-regulated in control plants and have not significant expression level in drought tolerance genotype.
(TIF)

S9 Fig The KEGG pathway analysis of each cluster was performed to assign the related biological pathways for each group of DEGs. Genes located in cluster I-VI were assigned to 31, 5, 2, 5, 16 and 37 KEGG pathways, respectively. Cluster I and II show enriched pathways which are down-regulated in drought tolerance genotype and control plants. Cluster III and IV comprised enriched pathways that related genes down and up-regulated with almost the same expression level across the comparisons. Cluster V and VI exhibited pathways which are up-regulated in drought tolerance genotype and control plants, respectively.
(TIF)
