# Supplementary figures and images for: Analysis of transcriptional responses in root tissue of bread wheat landrace (Triticum aestivum L.) reveals drought avoidance mechanisms under water scarcity
Source: PLoS One. 2019 Mar 6;14(3):e0212671. doi: 10.1371/journal.pone.0212671 (PMC6402654; doi:10.1371/journal.pone.0212671)

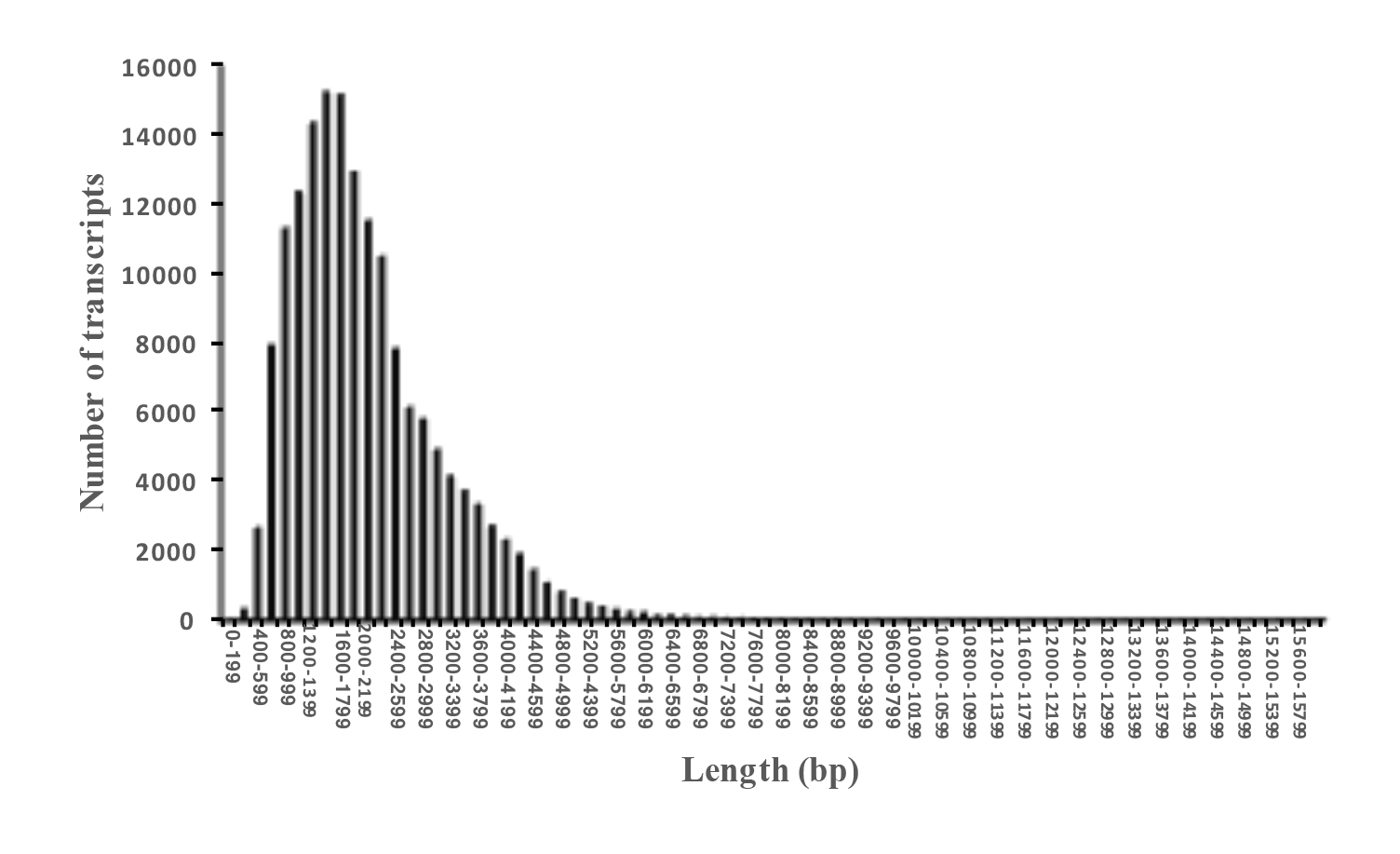

Supplement: S1 Fig — (TIF) [file pone.0212671.s002.tif]

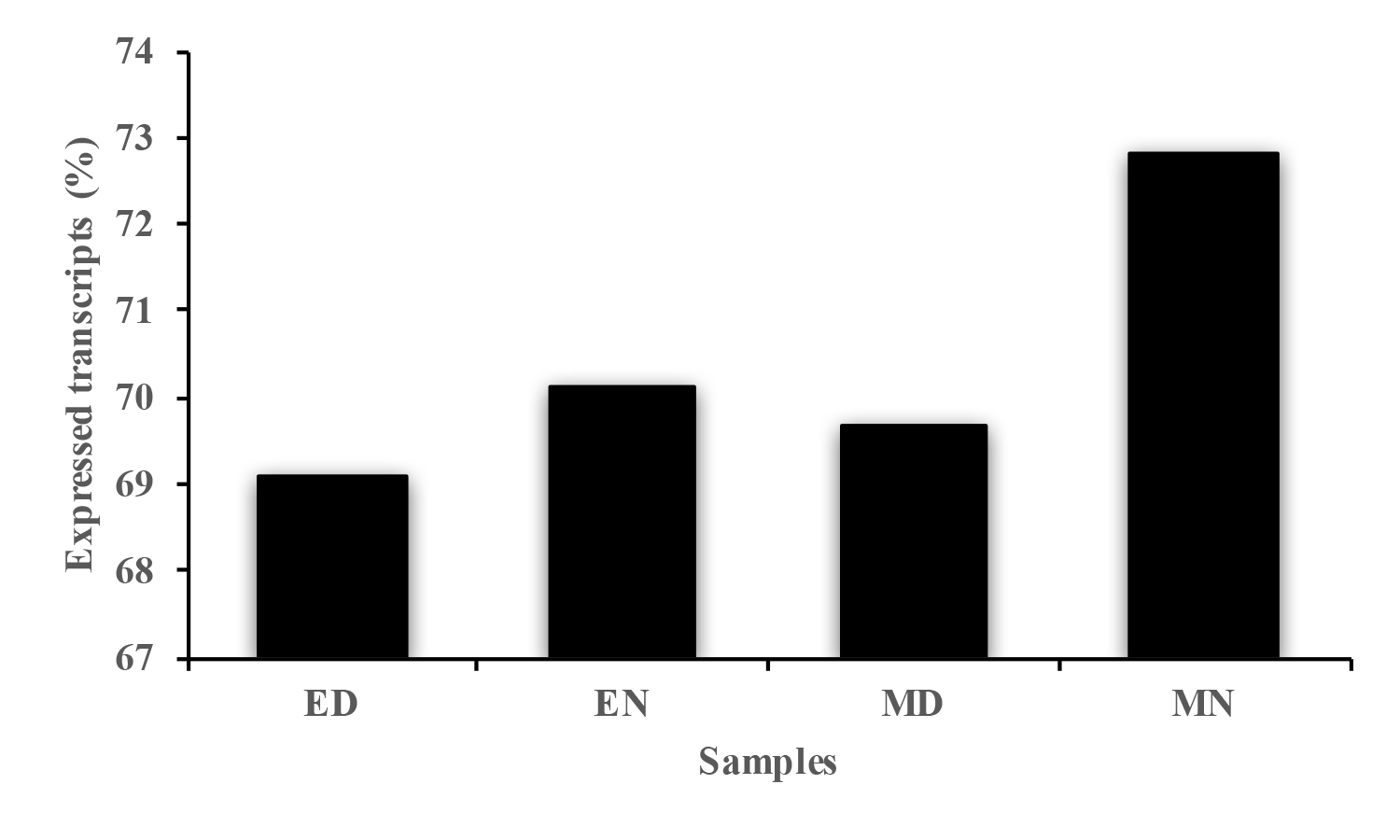

Supplement: S2 Fig — E and M represent “L-82” and “Marvdasht” genotype, respectively. D and N means drought stress and normal condition. (TIF) [file pone.0212671.s003.tif]

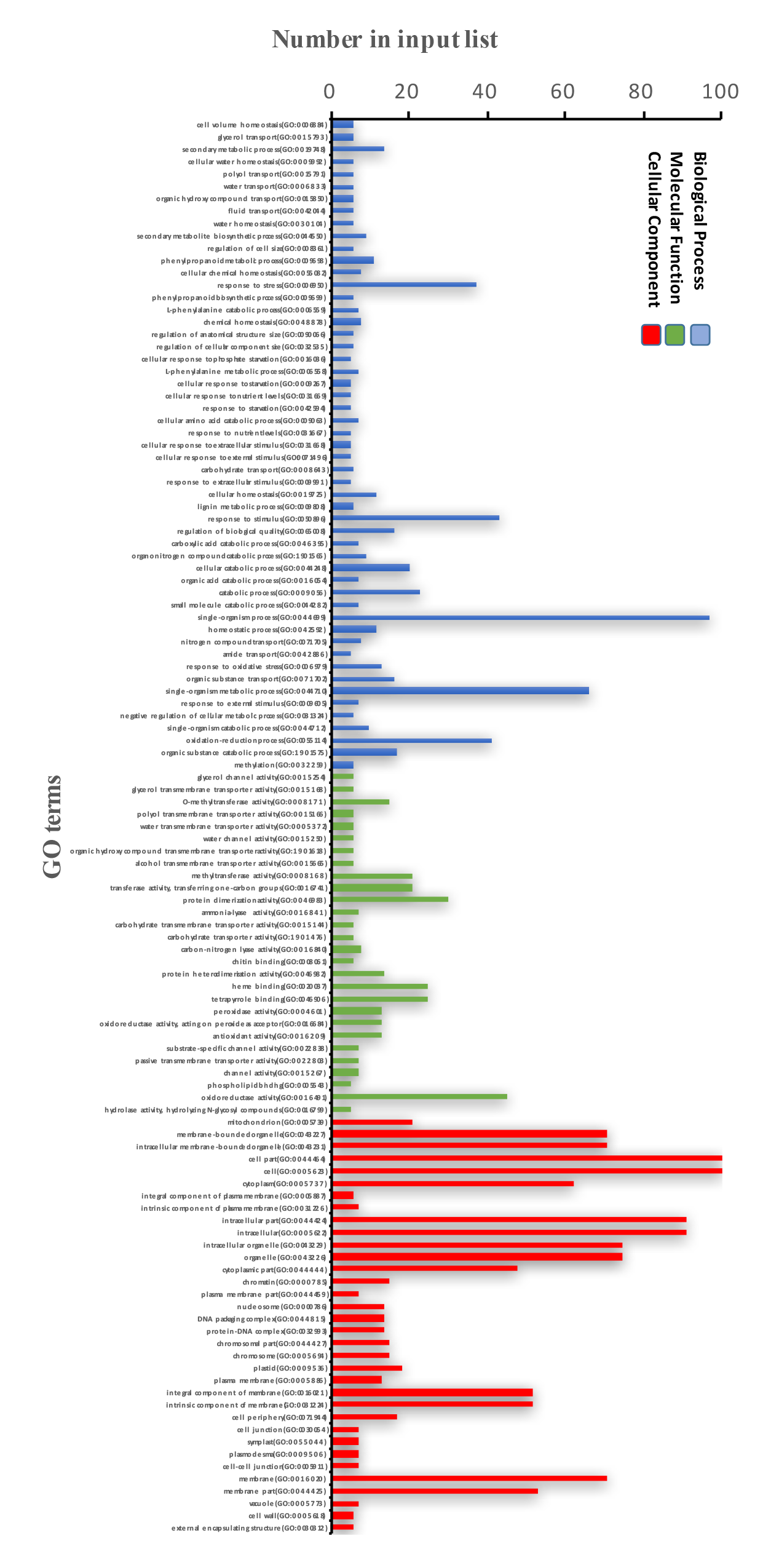

Supplement: S3 Fig — DEGs were analyzed using AgriGo and overrepresented terms in the three main categories “Biological Process”, “Molecular Function”, and “Cellular Component” were filtered using Fisher’s exact test and the Benjamini-Hochberg multiple testing correction (Q-value < 0.05). (TIF) [file pone.0212671.s004.tif]

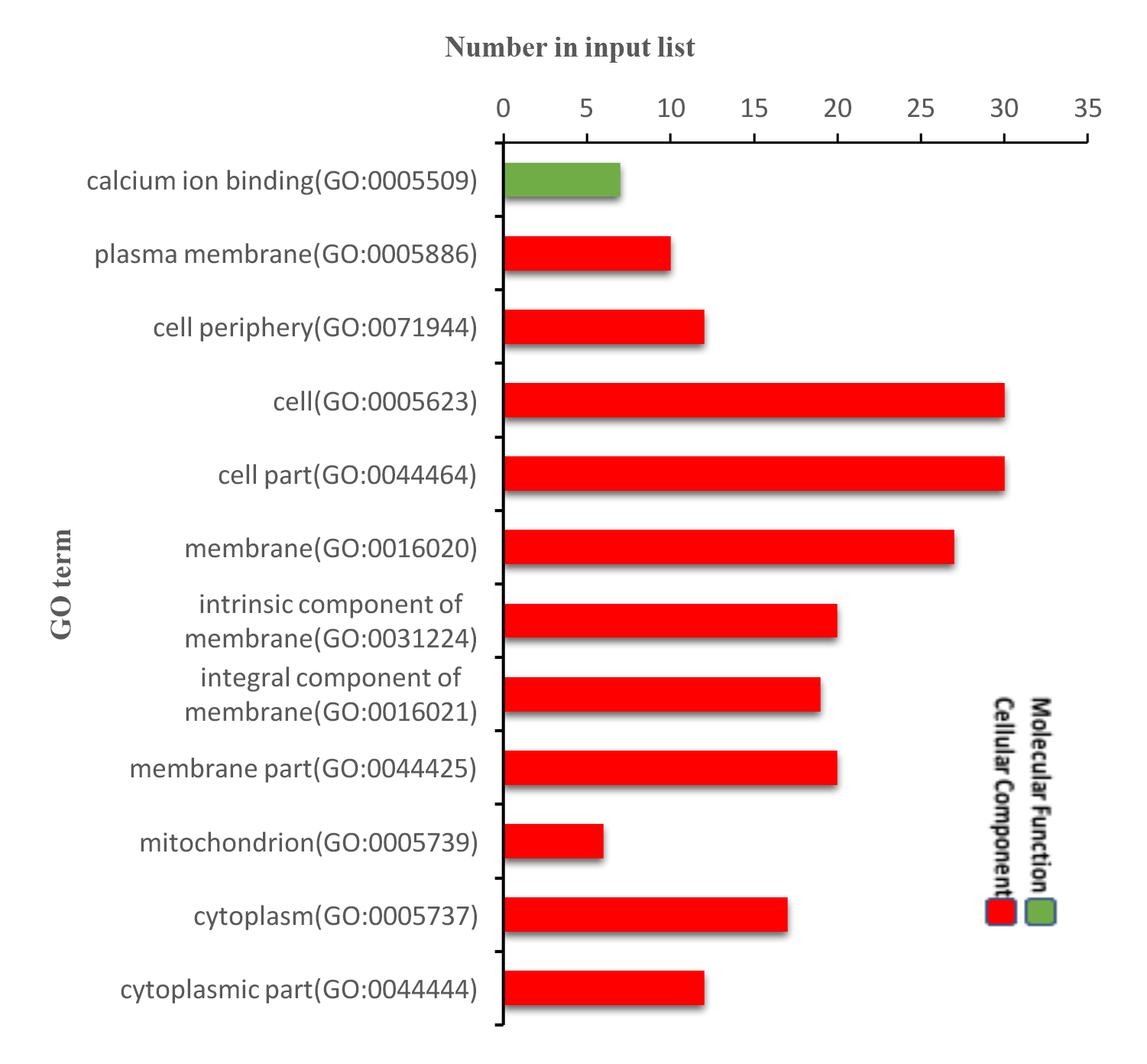

Supplement: S4 Fig — DEGs were analyzed using AgriGo and overrepresented terms in the three main categories “Biological Process”, “Molecular Function”, and “Cellular Component” were filtered using Fisher’s exact test and the Benjamini-Hochberg multiple testing correction (Q-value < 0.05). (TIF) [file pone.0212671.s005.tif]

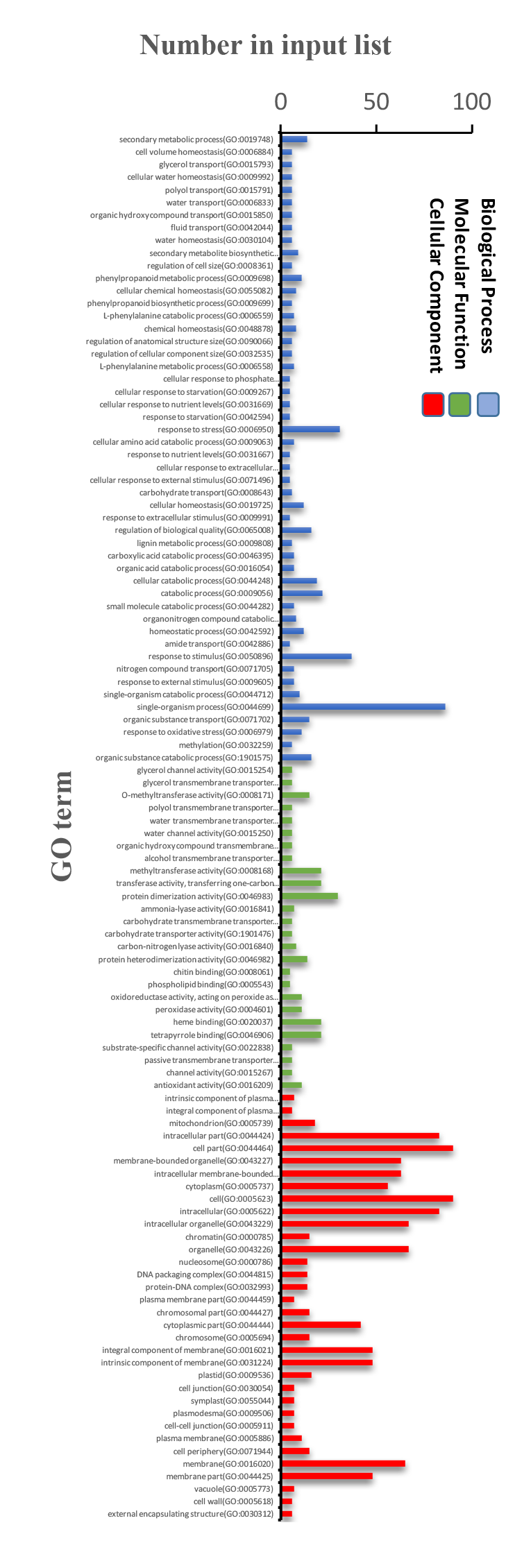

Supplement: S5 Fig — DEGs were analyzed using AgriGo and overrepresented terms in the three main categories “Biological Process”, “Molecular Function”, and “Cellular Component” were filtered using Fisher’s exact test and the Benjamini-Hochberg multiple testing correction (Q-value < 0.05). (TIF) [file pone.0212671.s006.tif]

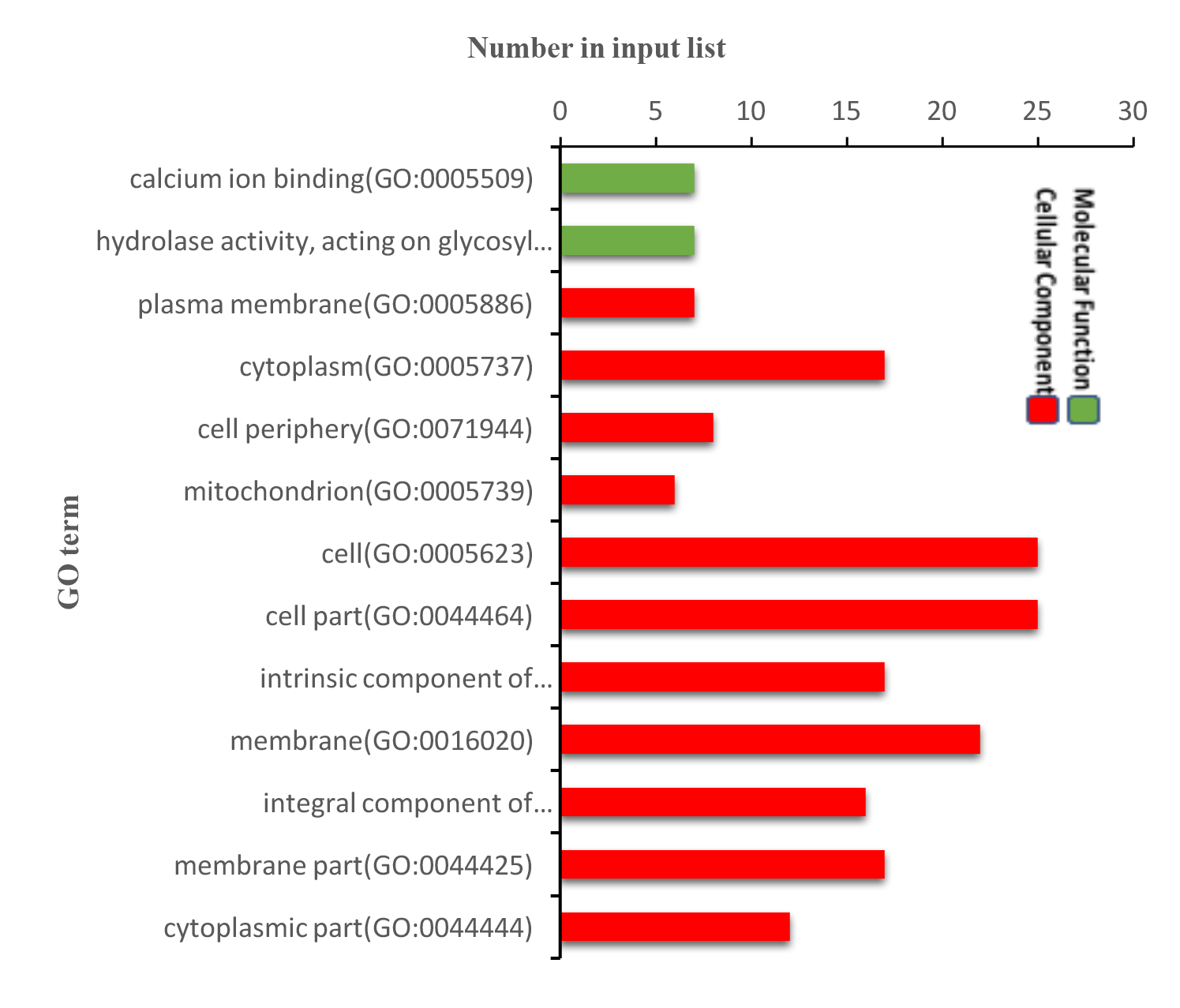

Supplement: S6 Fig — DEGs were analyzed using AgriGo and overrepresented terms in the three main categories “Biological Process”, “Molecular Function”, and “Cellular Component” were filtered using Fisher’s exact test and the Benjamini-Hochberg multiple testing correction (Q-value < 0.05). (TIF) [file pone.0212671.s007.tif]

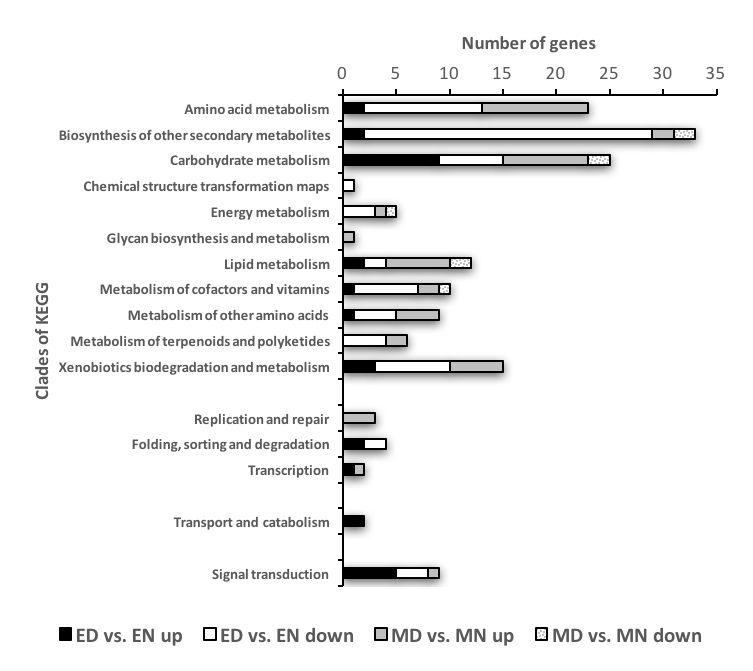

Supplement: S7 Fig — These different pathways were assign to 16 clades under four major KEGG categories namely, “metabolism”, “genetic information processing”, “cellular processes” and “environmental information processing”. (TIF) [file pone.0212671.s008.tif]

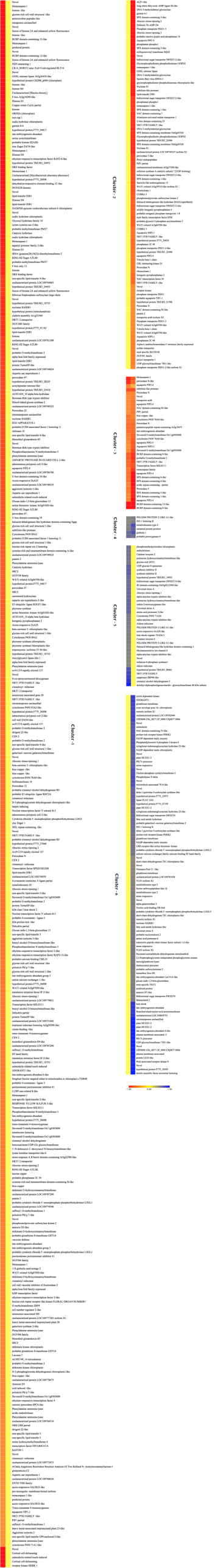

Supplement: S8 Fig — The genes in cluster I (382) down-regulated in drought tolerance genotype while had no differential expression in control plants. Cluster II had genes (96) that down-regulated in control plants but have not differential expression in drought tolerant genotypes. Cluster III (35) and IV (6) comprised down and up-regulated genes with almost the same expression profile across the comparisons. Cluster V (37) exhibited DEGs which are up-regulated in drought tolerance genotype while had no differential expression in control plants. Cluster VI shows genes (99) that are up-regulated in control plants and have not significant expression level in drought tolerance genotype. (TIF) [file pone.0212671.s009.tif]

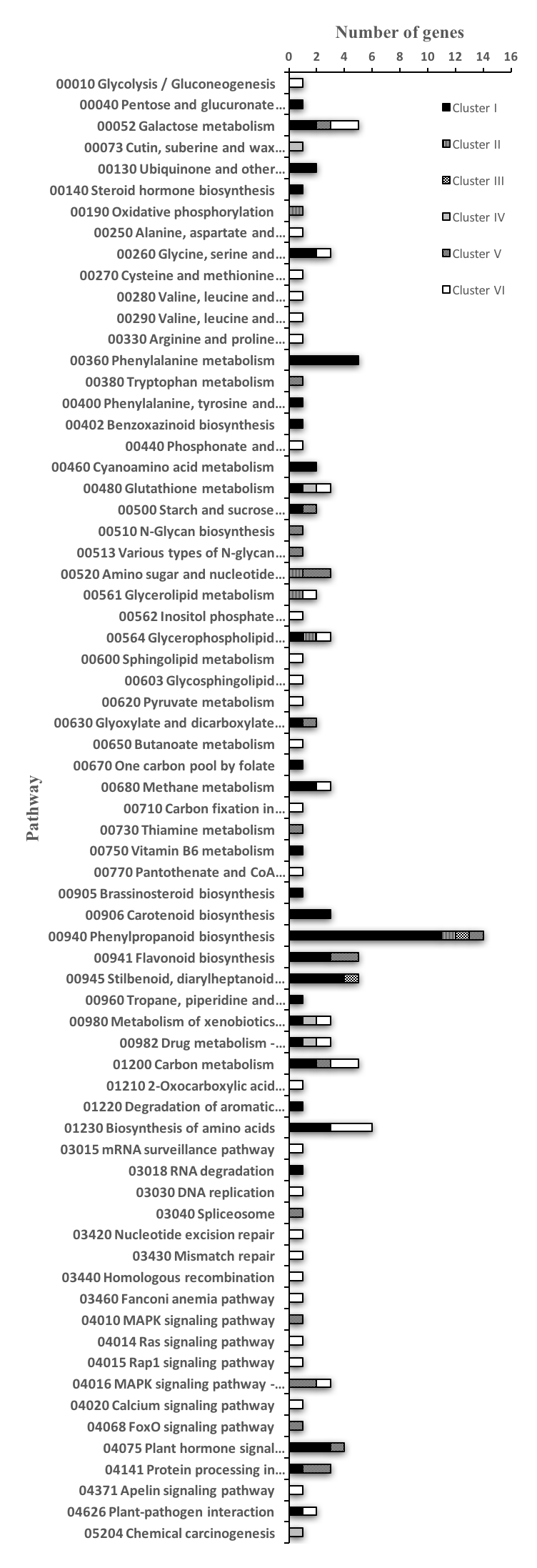

Supplement: S9 Fig — Genes located in cluster I-VI were assigned to 31, 5, 2, 5, 16 and 37 KEGG pathways, respectively. Cluster I and II show enriched pathways which are down-regulated in drought tolerance genotype and control plants. Cluster III and IV comprised enriched pathways that related genes down and up-regulated with almost the same expression level across the comparisons. Cluster V and VI exhibited pathways which are up-regulated in drought tolerance genotype and control plants, respectively. (TIF) [file pone.0212671.s010.tif]
